# A Case of Lyme Carditis Presenting with Atrial Fibrillation

**DOI:** 10.1155/2018/5265298

**Published:** 2018-09-02

**Authors:** Peter J. Kennel, Melvin Parasram, Daniel Lu, Diane Zisa, Samuel Chung, Samuel Freedman, Katherine Knorr, Timothy Donahoe, Steven M. Markowitz, Hadi Halazun

**Affiliations:** ^1^Department of Medicine, Weill Cornell Medicine, New York, NY, USA; ^2^Department of Neurology, Weill Cornell Medicine, New York, NY, USA; ^3^Department of Cardiology, Weill Cornell Medicine, New York, NY, USA

## Abstract

We report a case of a 20-year-old man who presented to our institution with a new arrhythmia on a routine EKG. Serial EKG tracings revealed various abnormal rhythms such as episodes of atrial fibrillation, profound first degree AV block, and type I second degree AV block. He was found to have positive serologies for *Borrelia burgdorferi*. After initiation of antibiotic therapy, the atrial arrhythmias and AV block resolved. Here, we present a case of Lyme carditis presenting with atrial fibrillation, a highly unusual presentation of Lyme carditis.

## 1. Background

Lyme disease is the most commonly reported vector-borne illness in the United States [1]. In 2016, 26,203 confirmed diagnoses of Lyme disease were reported [2]. The incidence of Lyme disease has been consistent over the last decade at approximately 8 cases per 100,000 persons in the US with 95% of confirmed Lyme disease cases reported from only 14 states, mostly in the Northeast region of the US. The most common clinical presentation of Lyme disease is erythema migrans (70%), followed by arthritis (30%), Bell's palsy (9%), radiculoneuropathy (4%), and meningitis/encephalitis (2%). Only 1% present with cardiac manifestations [3]. Cardiac manifestations of Lyme disease are rare but well described. Lyme carditis (LC) occurs in 0.3–4% of untreated adults with Lyme disease in Europe and in 1.5–10% of untreated adult patients in the United States [4]. For reasons yet to be determined, there is a male predominance of 3 to 1 in LC [5]. Cardiac involvement occurs during the early, disseminated phase of the disease [5], and cardiac symptoms can develop between the 7th day and the 7th month (median 21 days) after the onset of erythema migrans [4, 6]. Pathophysiologically, myocardial biopsy specimens usually show transmural inflammatory infiltrates with characteristic endocardial lymphocytic infiltrates and occasionally spirochetes [7]. Most cases of LC appear to be clinically asymptomatic. If symptomatic, typical presentations may be complaints of fatigue, dyspnea, palpitations, lightheadedness, syncope, and chest pain [8]. LC usually has a benign clinical course with complete resolution of symptoms. However, serious and even fatal cardiac complications have been reported, including fatal cardiac arrhythmias, myocarditis, cardiac tamponade, heart failure, and sudden cardiac death [4]. The typical cardiac presentations are various degrees of atrioventricular block [8, 9]. Rarely, LC has been reported to cause isolated bundle branch blocks or other conduction abnormalities [4, 8, 10]. There are rare reports of ventricular and supraventricular arrhythmias associated with LC. A recent case reported by Frank et al. describes a new-onset junctional tachycardia in a pediatric case of LC [11]. In another recent publication, Wenger et al. report a case of an adult man presenting with atrial fibrillation, complete atrioventricular block, and escape rhythm attributed to LC [12]. Here, we report a case of a young adult man with Lyme disease presenting with atrial fibrillation, profound first degree AV block, and type I second degree AV block.

## 2. Case Presentation

**Figure 1:**
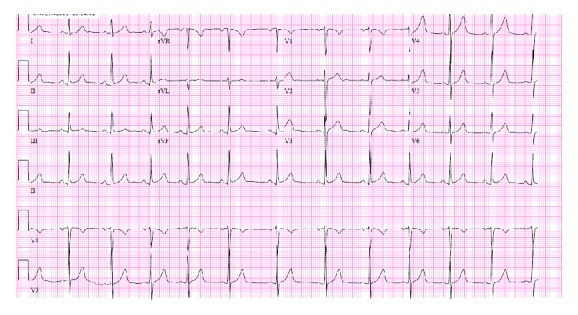
Baseline EKG. Normal sinus rhythm with a short PR interval.

A 20-year-old man was referred to our institution from an oncological clinic where he was undergoing maintenance chemotherapy for metastatic alveolar rhabdomyosarcoma. In routine EKG, there was concerning new EKG abnormalities with a possible new AV block. Prior EKGs had been without abnormal findings (Figure 1). Given the new EKG changes, the patient was admitted to an outside hospital for observation. Prior to his admission, the patient had been asymptomatic and had unlimited exercise capacity. He denied any tick bites or rashes in the recent past but reported that he had been hiking over the summer in Orange County, NY.

The patient had a medical history of left forearm alveolar rhabdomyosarcoma, diagnosed 16 months prior to this presentation for which he had undergone radiation therapy to his arm and chemotherapy including irinotecan, carboplatin, vincristine, doxorubicin (cumulative dose 300 mg/m^2^; initial regimen, which had been completed), and a combination of cyclophosphamide, vinorelbine, and temsirolimus (maintenance chemotherapy regimen). Recent imaging including PET had shown no evidence of disease, and the patient was deemed to be in remission at the time of presentation. The patient's baseline EKG prior to his presentation showed a normal sinus rhythm with a PR interval of 152 msec (Figure 1).

The patient's home medications were sulfamethoxazole and trimethoprim prophylaxis, cyclophosphamide, and zolpidem. He was a lifetime nonsmoker, did not consume alcohol or illicit drugs, and lived with his family with no cardiac family history.

**Figure 2:**
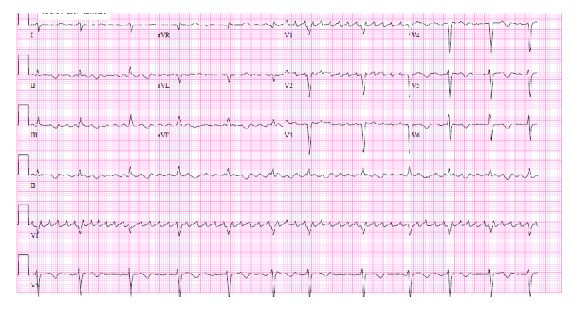
EKG on admission shows coarse atrial fibrillation with a slow ventricular response. Whereas the tracings in the precordial leads to appear organized, the tracing in lead II suggests coarse atrial fibrillation.

On arrival to our institution, the patient was asymptomatic. His blood pressure was 108/63 mmHg, heart rate was regular and between 80 and 115 bpm, he was afebrile at 36.4°C, and his oxygen saturation was 100% on room air. His physical exam was unremarkable, with no cardiopulmonary findings, no focal neurological deficits, and no abnormal skin findings. The initial EKG on admission revealed coarse atrial fibrillation with a ventricular rate of 60 beats per minute (Figure 2).

Initial laboratory results were only notable for a hemoglobin of 10.0 mg/dl, a mild relative lymphocytopenia with a normal white blood cell count. TSH and troponin I levels were within normal limits. Echocardiogram showed normal left and right heart function with no wall motion abnormalities, mild tricuspid valve regurgitation, and no pericardial effusion.

**Figure 3:**
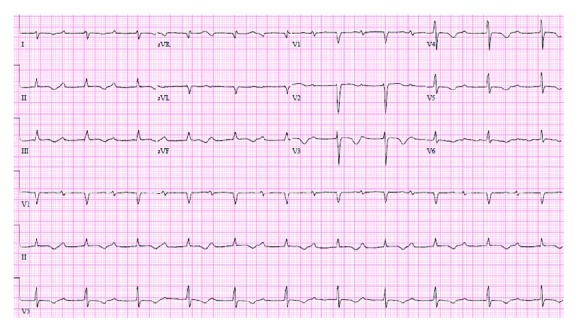
Profound PR prolongation.

A cardiac MRI, performed 2 days after presentation, showed mild right atrial dilatation and no other abnormalities. In particular, no signs of inflammation or masses were found. Additionally, on hospital day 2, telemetry monitoring and EKG revealed spontaneous conversion to normal sinus rhythm with a profoundly prolonged PR interval of 460 msec (Figure 3).

Further workup during the hospital course revealed a twice positive *B. burgdorferi* IgG and IgM immunoblot (performed at ARUP Laboratories; IgG: bands present: 66, 45, 41, 39, 23, and 18 kDa, IgM: bands present: 41 and 39 kDa).

## 3. Treatment

**Figure 4:**
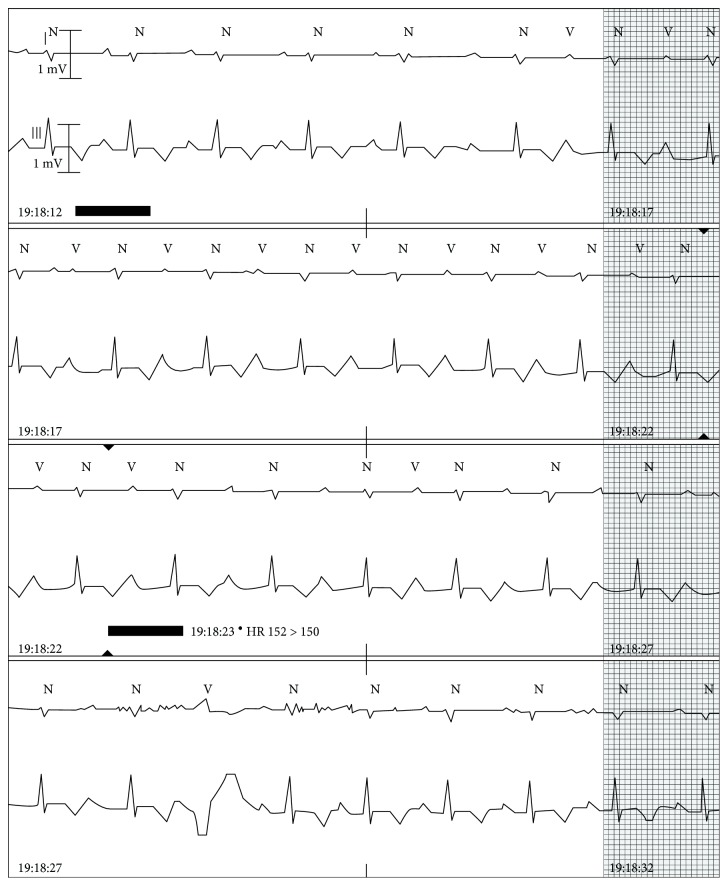
Abrupt change of PR intervals. Abrupt shift from relatively short PR to long PR (first line) and recurrent shift from long PR to shorter PR (last line). This second shift occurs after a PVC, which is a known phenomenon in which the PVC resets conduction to allow the fast pathway to conduct on the next beat.

Given the positive Lyme serology in the setting of a new AV block and atrial fibrillation, antibiotic therapy with intravenous ceftriaxone (2 grams every 24 h) was initiated. The patient was monitored with daily EKGs and continued telemetry monitoring. Telemetry showed intermittent episodes of abrupt changes from relatively short to long PR intervals.  Figure 4 shows an abrupt spontaneous shift from short to long PR. Abrupt shortening of the PR interval occurred following a late-coupled spontaneous premature ventricular complex (PVC) consistent with retrograde concealed conduction into the AV node facilitating antegrade fast pathway conduction on the subsequent beats.

On continued antibiotic therapy, the patient remained in sinus rhythm with shortening PR intervals to 390 msec on the day of discharge. The patient was discharged with a 21-day course of home IV ceftriaxone, a home telemetry monitor, and close follow-up with our electrophysiology clinic.

## 4. Outcome

**Figure 5:**
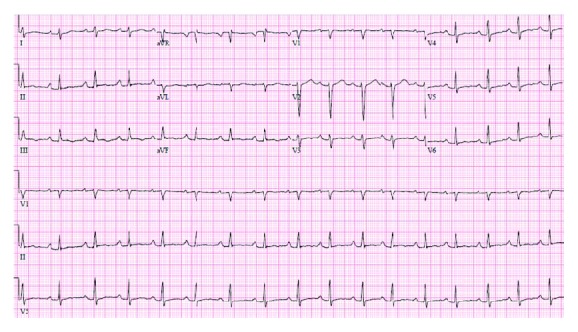
After antibiotic therapy, resolution of AV block.

At home, an event monitor for 1 week showed no advanced AV block or profound bradycardia. Recorded events were a HR 53 to 137 bpm, average 85 bpm, 147 supraventricular runs up to 33 beats; no advanced AV blocks were recorded. Over the course of his antibiotic therapy, the PR interval on serial EKG recordings shortened from 460 msec on admission to 184 msec after 3 weeks of intravenous ceftriaxone, without any evidence of episodes of higher degree AV blocks (Figure 5). At the end of his treatment, the PR intervals were consistently short.

## 5. Discussion

Here, we report a case of a young adult man with Lyme disease presenting with atrial fibrillation, profound first degree AV block, and type I second degree AV block. This is an unusual presentation of LC, in which AV block is a known entity; however, atrial fibrillation is highly unusual. Lyme disease is an overall rare disease with only around 26,000 cases reported in the US per year. Only around 4% of these cases will develop LC. Among these patients, the vast majority experiences some degree of AV block. To our knowledge, there are only anecdotal reports of LC presenting with supraventricular arrhythmias as we describe in the case at hand. Of note, the patient also had alterations in his PR interval, sometimes occurring from one beat to the next. This pattern suggests the presence of dual AV nodal physiology whereby abrupt PR prolongation occurs following antegrade block in the fast pathway. Dual atrial inputs are likely to be an intrinsic property of the AV node, but this manifestation of abrupt PR changes is rarely seen in normal individuals. Interestingly, this phenomenon has been reported as a manifestation of Lyme disease in another case report [13]. Decremental physiology of the fast pathway is favored by gradual PR prolongation until block occurs in the fast pathway and conduction proceeds down a slow AV nodal pathway. It is possible that Lyme carditis preferentially affected particular components of the AV node associated with the fast pathway.

While the patient was diagnosed with LC, a rare presentation of a rare disease, his past medical history was also notable for ARMS, a rare pediatric malignancy, which has an incidence of around 1 per 1 million. Notably, the patient was diagnosed with stage 4 disease with an overall poor prognosis, but underwent an experimental radio-chemotherapeutic protocol and was deemed free of disease after completion of the treatment. Given his prior exposure to chemotherapeutic drugs, cardiotoxic effects of these agents needed to be reviewed as possible causes for his new-onset arrhythmias: the experimental chemotherapeutic regimen consisted of irinotecan, carboplatin, vincristine, cyclophosphamide and vinorelbine, and doxorubicin (Clinical Trials Identifier NCT00077285). While the aforementioned drugs are not associated with cardiotoxic effects, doxorubicin is known to be cardiotoxic and can cause atrial fibrillation [14]. However, atrial fibrillation would usually present as an acute cardiotoxic effect and unlikely as delayed cardiotoxicity. The patient had completed the chemotherapeutic regimen containing doxorubicin (cumulative dose 300 mg/m^2^) around a year prior to the onset of atrial arrhythmias, and MRI and echocardiogram did not show signs of myocarditis or heart failure. Furthermore, the patient was exposed to a low cumulative dose of doxorubicine. Cardiac complications at comparable doses have been found to be as low as 0–2.8% [15]. Hence, it appears unlikely that the chemotherapeutic agents contributed to the cardiac findings.

Other rare differential diagnoses to consider in this case may be other causes for myocarditis or cardiomyopathies, familial syndromes such as Lenegre disease, cardiac tumors given his history of rhabdomyosarcoma, or syndromes in the rheumatologic realm. However, an extensive workup including cardiac MRI showing no evidence of structural abnormalities, signs of inflammation, or heart failure, repeat echocardiogram, and serologic studies including troponin, RPR, and toxoplasmosis remained unrevealing.

Our case report is limited in that we are unable to conclusively identify the cause of the new-onset atrial fibrillation in this patient as, even though serologies and response to antibiotic treatment hint towards LC being the cause for the arrhythmia, other causes cannot be excluded. Of note, given the finding of mild atrial dilation on cardiac MRI, it is possible that LC could have been a “second hit” in the setting of a preexisting abnormal substrate.

In summary, LC needs to be considered as a differential diagnosis in patients presenting with new-onset supraventricular arrhythmias if they may have been exposed to vectors of borrelia or live in endemic areas.
